# Successful Outcome in a Geriatric Patient With Giant Dedifferentiated Liposarcoma of the Lower Extremity: A Case Report

**DOI:** 10.7759/cureus.87800

**Published:** 2025-07-12

**Authors:** Javier Martinez Martinez, Mauricio Munoz Munoz, Carlos O Pacheco Gonzalez

**Affiliations:** 1 General Surgery, Hospital General Dr. Norberto Treviño Zapata, Ciudad Victoria, MEX; 2 Oncologic Surgery, Hospital General Dr. Norberto Treviño Zapata, Ciudad Victoria, MEX

**Keywords:** dedifferentiated liposarcoma, geriatric patient, giant liposarcoma, surgical case report, surgical oncology

## Abstract

Liposarcomas are rare malignant soft tissue tumors; the dedifferentiated liposarcoma (DDL) subtype accounts for 6% of all soft tissue sarcomas. This tumor can develop de novo or transition from an atypical lipomatous tumor (ALT), occurring more frequently in the retroperitoneum; presentation in the extremities is uncommon. This subtype behaves aggressively, leading to local recurrence, metastasis, and a poor disease-free survival rate if treated improperly. We present the case of a 70-year-old female with a history of a giant soft tissue mass of one year of growth, with limited range of motion of her right lower extremity and edema. Initial histological analysis identified the mass as a well-differentiated liposarcoma/ALT. Post-surgical histological examination revealed a DDL, measuring 28 cm x 24 cm x 21 cm. Dedifferentiation from a well-differentiated tumor happens in approximately 10% of cases. Age, location, size, histological subtype, and presence of metastasis are the most important prognostic factors. Early suspicion of liposarcoma is paramount when facing a large, growing tumor in soft tissues; histological examination must be done to offer the proper treatment.

## Introduction

Soft tissue sarcomas are rare, heterogeneous, and malignant tumors. They account for 1% and 7% of all adult and pediatric tumors, respectively [1-2]. The World Health Organization (WHO) classifies soft tissue sarcomas in 11 different categories. Liposarcomas are listed as malignant adipocytic tumors, and they are divided into five histological subtypes: well differentiated, dedifferentiated, myxoid, pleomorphic, and myxoid pleomorphic [3]. Liposarcomas are the most frequent malignant soft tissue tumors, accounting for 20% of all soft tissue sarcomas. They can appear anywhere in the body; however, they have a predilection for the retroperitoneum, followed by head, neck, trunk, extremities, and spermatic cord [4-6]. Dedifferentiated liposarcoma (DDL) is a high-grade and aggressive tumor. This subtype presents as a transition from a previous atypical lipomatous tumor (ALT) in 10% of cases; the remaining 90% occur de novo. DDL accounts for 6% of all soft tissue sarcomas, does not have gender predilection, more often present in elderly patients between the sixth to seventh decades, symptoms usually are related to the anatomic location, frequently referred as a painless, large mass characterized by slow growth through several years [7-8]. Most soft tissue tumors tend to be benign; however, any soft tissue mass larger than five cm, located in a deep site, associated with pain or any skin changes, within a short period, must be studied according to the latest United Kingdom guidelines for soft tissue sarcomas [9].

Genetic amplifications of the *murine double minute 2* (MDM2) and *cyclin-dependent kinase 4 *(CDK4) oncogenes have been associated with the presentation of DDL. These amplifications are shared with well-differentiated liposarcoma (WDL); however, DDL has a more aggressive behavior than WDL [10-11]. Diagnosis is made through histological examination; it may occur as a transition between well-differentiated areas to dedifferentiated ones. The spectrum of dedifferentiation is variable. Microscopically, WDL consists of mature adipocytes with nuclear atypia and hyperchromatic stromal spindle cells, whereas DDL typically exhibits discrete non-lipomatous areas with a wide morphological spectrum usually resembling undifferentiated pleomorphic sarcoma or fibrosarcoma, making the transition from a WDL to non-lipomatous sarcoma the histological hallmark of DDL [12]. Molecular testing, like fluorescence in situ hybridization (FISH), is a very useful tool to identify DDL through amplification of MDM2 and CDK4 [13]. Magnetic resonance imaging (MRI) is preferred for studying soft tissue sarcomas; this diagnostic tool helps us identify size, location, and any relationship to adjacent structures [14]. DDL in extremities is uncommon, usually accounting for 25% of cases [15]. Age, location, size, histological subtype, and presence of metastasis are the most important prognostic factors [16-17]. Surgery is the core treatment when we face a localized tumor; the challenge is to remove the tumor completely without causing morbidity and securing functional results. DDL is known to have an aggressive clinical course with high local recurrence rates of 44% and metastatic rates of 40% [18]. The most common metastatic sites are pulmonary, hepatic, extrapulmonary, and extrahepatic. When metastatic disease is established, the first-line treatment is systemic therapy, such as doxorubicin, doxorubicin-ifosfamide, or selinexor, which only offers palliative treatment [19].

## Case presentation

**Figure 1:**
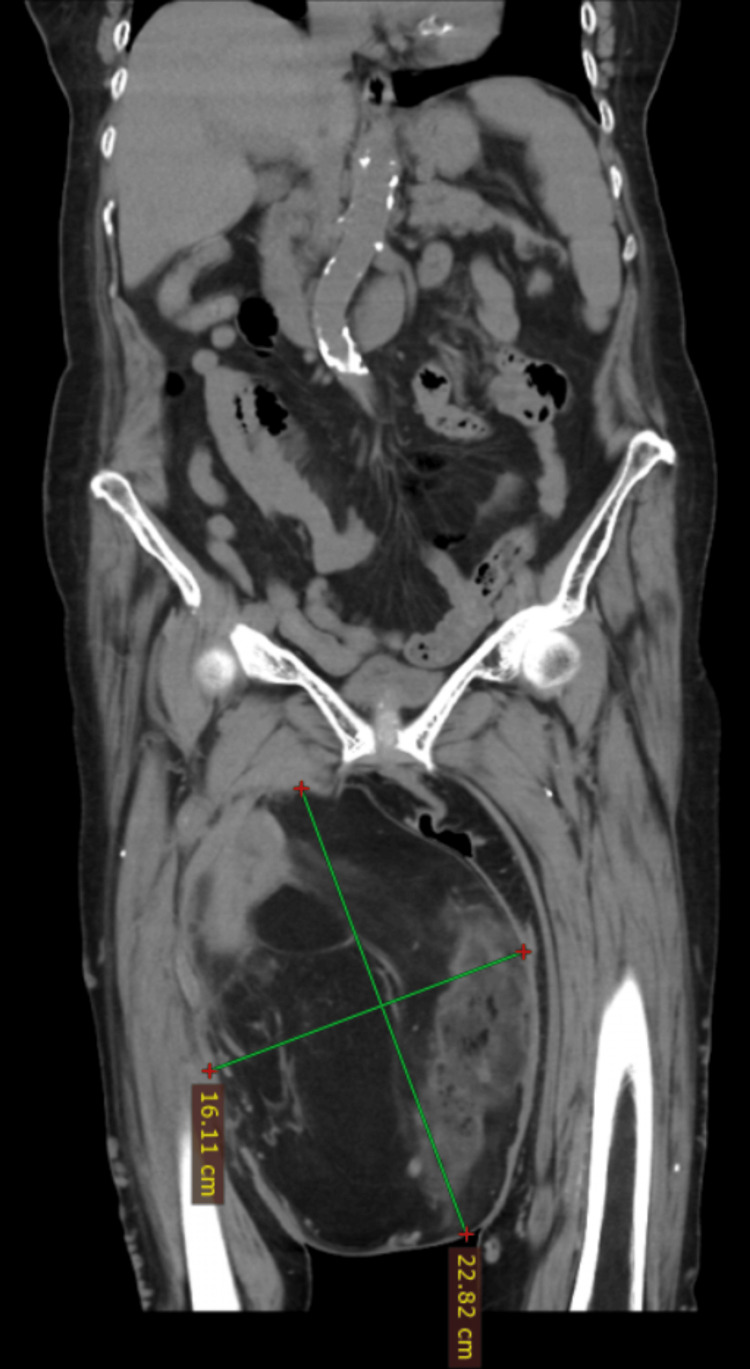
Coronal CT image showing a giant solid mass with areas of hypo- and hyperdensity.

**Figure 2:**
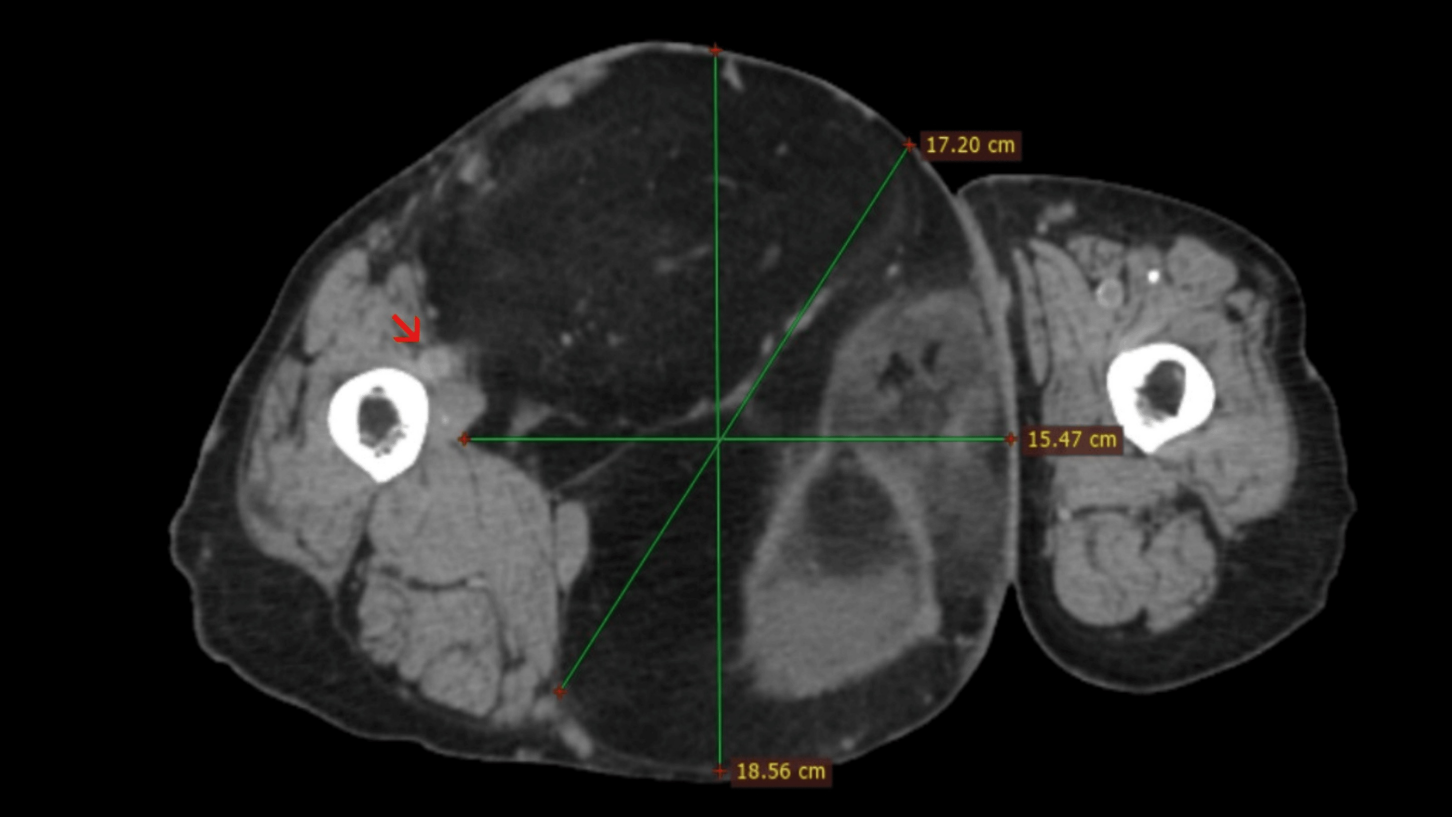
Axial CT image showing a giant solid mass in the right anterior thigh without involvement of vascular structures (right arrow).

A 70-year-old female with past medical history of high blood pressure and exposure to firewood over 40 years, arrived at our healthcare unit, a second level hospital in public setting, referring a giant mass in her right lower extremity, with a year of growth associated to edema and limited range of motion of the extremity. Physical exploration revealed a large mass located in the right anterior thigh, painless with telangiectasias and an ulceration in the center, with no signs of local infection. Diagnostic workup followed an incisional biopsy; the first histological examination revealed a WDL/ALT. After histological diagnosis was made of a soft tissue sarcoma, the patient was referred to the oncologic surgery department for her treatment. Due to limitations at our healthcare unit, a computed tomography (CT) scan was ordered, which revealed a giant soft tissue mass measuring 22 × 16 cm, without involvement of vascular structures (Figures 1-2).

**Figure 3:**
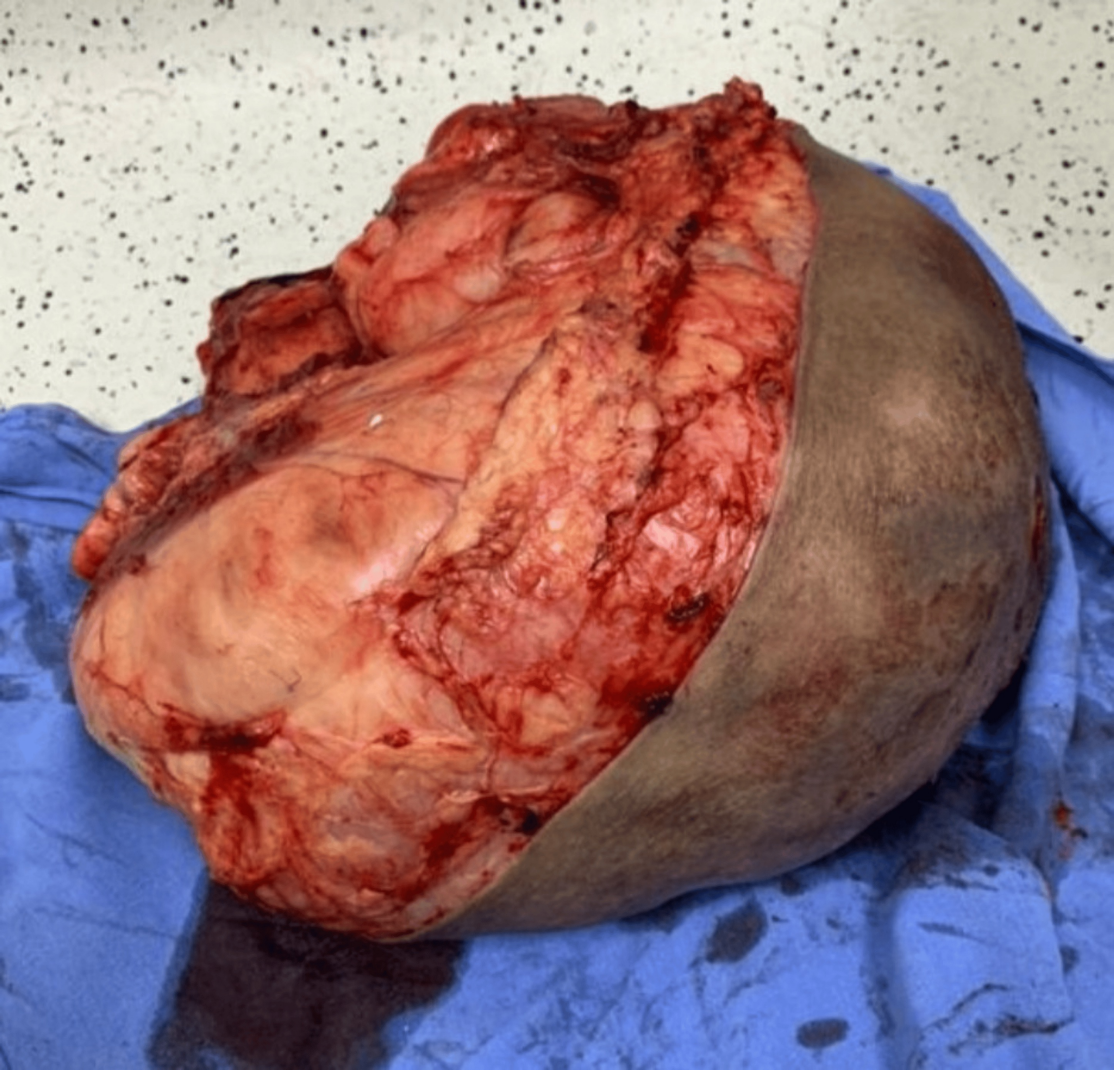
Resected surgical specimen measuring 28 × 24 × 21 cm, including surrounding muscle and areas of ischemic and necrotic tissue.

**Figure 4:**
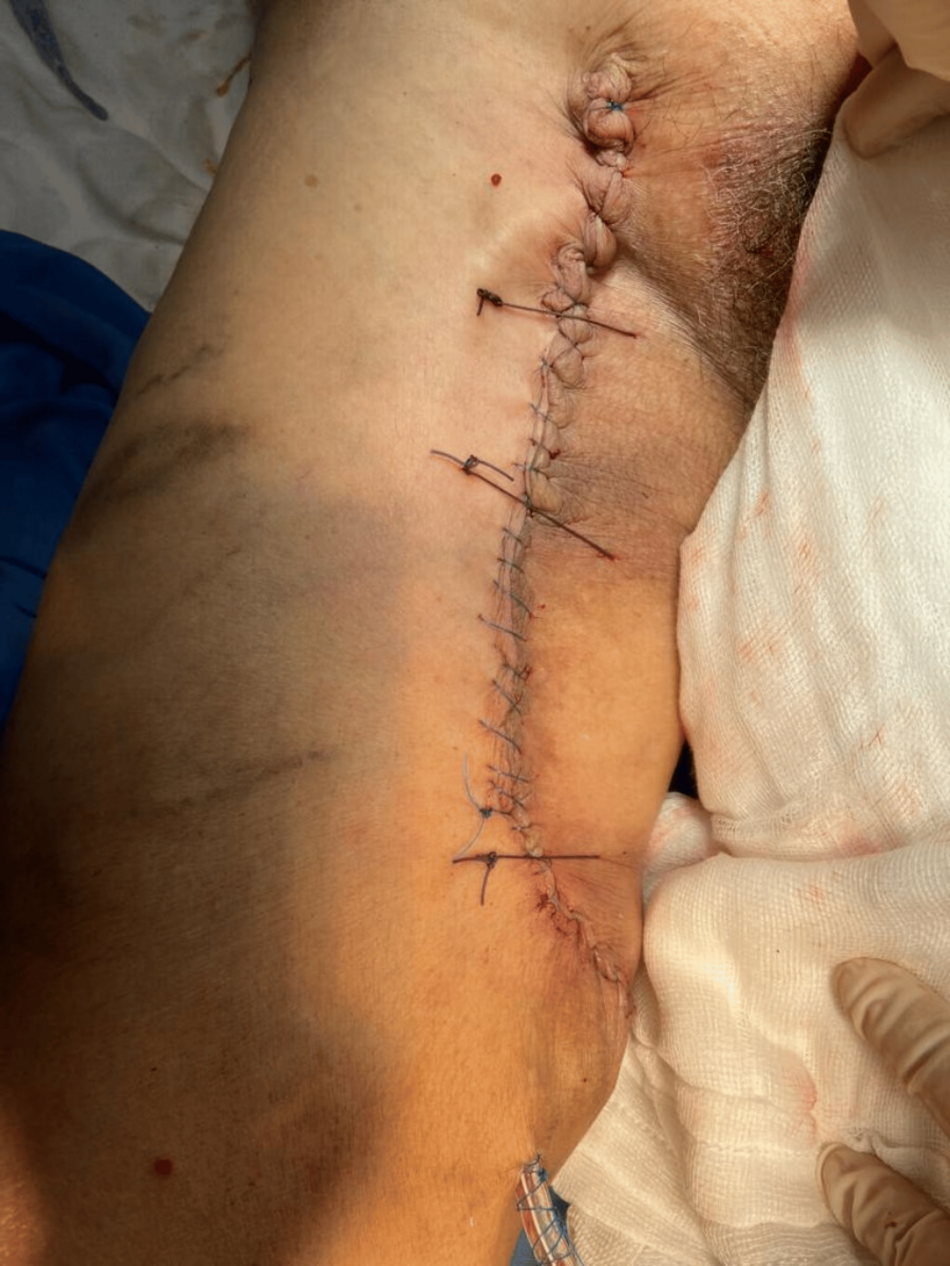
Primary closure was achieved with placement of a closed drainage system.

**Figure 5:**
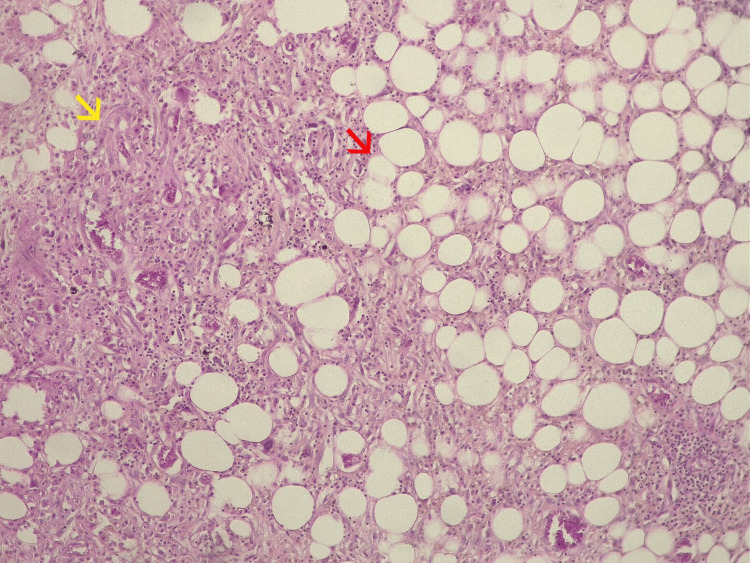
Dedifferentiated liposarcoma showing a transition from a well-differentiated lipogenic component (red arrow) to a dedifferentiated component composed of spindle-shaped cells with a fascicular growth pattern (yellow arrow).

Elective surgery was performed 18 weeks later by the chief oncologic surgeon. A wide resection was carried out without neurovascular injury, and the surgical margins included surrounding muscle and areas of ischemic and necrotic tissue (Figure 3). Due to the large growing mass, there was sufficient skin and soft tissue for a primary closure, and a closed drainage was placed (Figure 4). Postoperative histological examination revealed a DDL measuring 28 × 24 × 21 cm, pseudo-encapsulated and lobulated, with involvement of striated muscle and an overlying skin ulcer. Resected margins and the surgical bed were free of cancer; no other therapeutic strategies were needed at this point. Microscopic description revealed a mesenchymal neoplasm consisting of medium-sized cells with a large nucleus, myxoid background, alternating with more solid areas of spindle-shaped cells, moderate nuclear pleomorphism, and atypical mitoses (Figure 5). Our patient was discharged home two days after her surgery, and drainage was removed seven days later, without postoperative complications. Again, due to limitations at our healthcare unit, neither an MRI nor a CT scan was available, so a chest X-ray was performed six months later, with no signs of metastasis observed. Follow-up during 18 months with no signs of local recurrence or distant metastasis. The mobility and strength of her right lower extremity improved and remained functional, with preserved sensitivity. Her only complaint was edema in the right lower extremity compared to the contralateral side.

## Discussion

DDL represents 6% of all soft tissue sarcomas. They appear more frequently in the retroperitoneum, followed by the head, neck, and trunk; location in the extremities is rare, with the lower extremities being more likely involved. Presentation usually involves middle-aged or elderly patients, peak incidence between 60 and 70 years old, which fits in the presentation of our patient; there’s no gender predilection reported in the literature. Symptoms are usually related to the anatomic location. In extremities, a growing painless mass is usually visible; any skin changes or a fast growth pattern are signs of alarm. All of the mentioned symptoms were reported by our patient. 

For most soft tissue sarcomas, etiology is unknown; DDL is not the exception, however. In the last decades, amplification of MDM2 and CDK4 oncogenes has been associated with this subtype. According to the latest radiology guidelines for soft tissue sarcomas, contrast-enhanced MRI is the main imaging tool for extremities, as it allows optimal evaluation of tumor extension and its relationship with surrounding muscle or neurovascular structures, very helpful for deciding the therapeutic strategy. However, due to the absence of an MRI in a second-level public health facility, only a CT scan was available for preoperative evaluation. Despite this limitation, it helped us rule out the involvement of neurovascular structures. FISH amplification was also unavailable at any public facility in our state.

A definitive diagnosis was made through histological examination. We first made an incisional biopsy four months before her surgery, which reported a WDL/atypical lipomatous tumor. A second histological examination of the en bloc resection confirmed a DDL, consistent with the 10% of cases in which a transition between histological patterns is observed. This is a great example to point out the time when differentiation between patterns occurred. Four months is a short period; as reported in the literature, the behavior of this subtype is aggressive.

Prognostic factors include age, tumor size, superficial or deep location, type of surgery, completeness of resection, local relapse, and presence of metastasis. Positive margins significantly increase the risk of local recurrence and decrease the disease-free survival rate.

Surgery remains the standard treatment for all adult-type localized soft tissue sarcomas. The goal is en bloc resection with R0 margins, which involves removing the tumor as a single specimen with a rim of surrounding normal tissue. Radiotherapy (RT) is typically used as standard treatment for high-grade lesions; however, its use should be discussed by a multidisciplinary team, taking into account risk factors, surgical margins, morbidity, and tumor size. RT is not indicated when a complete resection with R0 margins can be achieved. Preoperative planning is essential to determine whether the patient could benefit from preoperative RT.

In our patient, the initial histological examination revealed a WDL, which has less aggressive behavior compared to a DDL. For this reason, preoperative RT was not considered. After surgical treatment, a different histological pattern was identified; however, given the nature of the surgery performed, we opted for observational management in accordance with the latest National Comprehensive Cancer Network (NCCN) guidelines for soft tissue sarcoma.

We achieved a single surgical resection with R0 margins while preserving the patient’s lower extremity, despite the large tumor size, thereby reducing morbidity. Follow-up over 18 months post-surgery revealed no signs of local recurrence or pulmonary or hepatic metastases.

## Conclusions

DDL is one of the most aggressive subtypes of liposarcoma; presentation in the extremities is not a common setting, and if not identified during early stages, they can evolve into a disease where we cannot offer definitive treatment. As we remain alert to clinical changes and maintain a high level of suspicion for this tumor, we can provide appropriate and effective treatment to our patients, reducing the morbidity and mortality associated with this disease. Surgery for localized liposarcoma is the mainstay of treatment, and when performed by a surgeon trained in soft tissue sarcomas, it offers a better prognosis.
